# Identification of *FZD4* and *LRP5* mutations in 11 of 49 families with familial exudative vitreoretinopathy

**Published:** 2012-10-04

**Authors:** Huiqin Yang, Shiqiang Li, Xueshan Xiao, Panfeng Wang, Xiangming Guo, Qingjiong Zhang

**Affiliations:** State Key Laboratory of Ophthalmology, Zhongshan Ophthalmic Center, Sun Yat-sen University, Guangzhou, P. R. China

## Abstract

**Purpose:**

To identify mutations in *FZD4* and *LRP5* in 49 Chinese families with familial exudative vitreoretinopathy (FEVR) and to reveal the mutation spectrum and frequency of these genes in the Chinese population.

**Methods:**

Clinical data and genomic DNA were collected for patients from 49 families with FEVR. The coding exons and adjacent intronic regions of *FZD4* and *LRP5* were amplified with polymerase chain reaction, and the resulting amplicons were analyzed with Sanger sequencing.

**Results:**

Eleven mutations were detected in 11 of the 49 families (22.4%), including five mutations in the *FZD4* gene in six families and six mutations in the *LRP5* gene in five families. Of the 11 mutations, eight were novel. Two families had the same *FZD4* mutation, and one family had compound heterozygous mutations in *LRP5*. The phenotypes of the patients with the mutations showed great variability.

**Conclusions:**

Our findings provide an overview of the mutation spectrum and frequency of *FZD4* and *LRP5* in Chinese patients with FEVR and emphasize the complexity of FEVR mutations and phenotypes.

## Introduction

Familial exudative vitreoretinopathy (FEVR, MIM 133780) is a hereditary disorder resulting from a developmental anomaly of the retinal vessels that may be stationary or progressive [1]. Patients with FEVR exhibit highly variable manifestations, ranging from asymptomatic to complete blindness. Progressive vascular anomalies impair vision due to various complications such as retinal neovascularization, exudates, fibrovascular proliferation, retinal folds, optic disc dragging, and retinal detachment [2]. Some minimally affected individuals may be detected only with fluorescein angiography of the peripheral retina, which exhibits avascularization and a nonperfusion zone [3].

Mutations in at least four genes have been identified as responsible for autosomal dominant (the *FZD4*, *LRP5,* and *TSPAN12* genes) [4–7], autosomal recessive (the *LRP5* gene) [8], or X-linked (the *NDP* gene) [9,10] FEVR. The encoded proteins of these four genes are involved in the wingless (Wnt) signaling pathway, which monitors retinal vascular development [7,11–14]. To date, several mutations have been identified in the four genes in patients with FEVR [15]. However, such studies in Chinese patients are limited [16,17]. To better understand the molecular defects underlying FEVR in the Chinese population, we performed a mutation screening of *FZD4* and *LRP5* in 49 Chinese families with FEVR and identified mutations in 11 families.

## Methods

### Patients

Written informed consent in accordance with the guidelines of the Declaration of Helsinki was obtained from the participating individuals or their guardians before the clinical data and genomic samples were collected. Ethical approval was provided by the Internal Review Board of the Zhongshan Ophthalmic Center, China. Probands from the 49 families with FEVR were collected from our Pediatric and Genetic Eye Clinic, Zhongshan Ophthalmic Center. Of the 49, 15 had a familial history of FEVR, and 34 were isolated cases. The clinical diagnosis of FEVR was as previously described [18–21]. *TSPAN12* mutations in the 49 families were excluded with Sanger dideoxy sequencing as described previously [21].

### Genetic analysis

Genomic DNA was prepared from venous leukocytes. The primer sequences used to amplify the coding exons and the adjacent intronic sequences of *FZD4* and *LRP5* are listed in Appendix 1. Touchdown polymerase chain reaction was performed, with the annealing temperature commencing at 64 °C, then decreasing by 0.5 °C after each cycle for the first 15 cycles, and finally being maintained at 57 °C for the remaining 21 cycles. Sequencing was performed with an ABI BigDye Terminator Cycle Sequencing Kit, v3.1, using an ABI 3100 Genetic Analyzer (Applied Biosystems, Foster City, CA). The sequences from the patients and the consensus sequences from the NCBI human genome database (*FZD4*: NC_000011.9 for gDNA, NM_012193.2 for mRNA, and NP_036325.2 for protein; *LRP5*: NC_000011.9 for gDNA, NM_002335.2 for mRNA, and NP_002326.2 for protein) were aligned by using the SeqManII program of the Lasergene package (DNAstar, Madison, WI). Each variation was initially confirmed with bidirectional sequencing and then evaluated in 192 chromosomes from 96 normal controls. The mutations were described according to the recommendations of the Human Genomic Variation Society (HGVS).

### Information assessment of missense mutations

Nonsynonymous substitutions were further analyzed by using a set of programs aimed at predicting the effect of the substitution at the protein level:

Sequence alignments with protein orthologs were used to determine whether an amino acid at the mutation position was evolutionarily conserved or not. Substitutions at evolutionarily conserved positions/sites are more deleterious than those at evolutionarily unconserved positions [22].

#### Blosum62

Blosum62 is an amino acid substitution scoring matrix. Missense mutations had a lower fraction of nonconservative changes (negative blosum62 scores) compared with that predicted from randomly distributed nonsynonymous single nucleotide polymorphisms, suggesting that blosum62 values predict deleterious function [23].

#### PolyPhen

PolyPhen is a sequence homology–based online tool used to predict the functional impact of a substitution. PolyPhen predicts how damaging a particular variant may be by using a set of empirical rules based on sequence, phylogenetic, and structural information about a particular variant [24].

## Results

### Mutations detected

**Figure 1:**
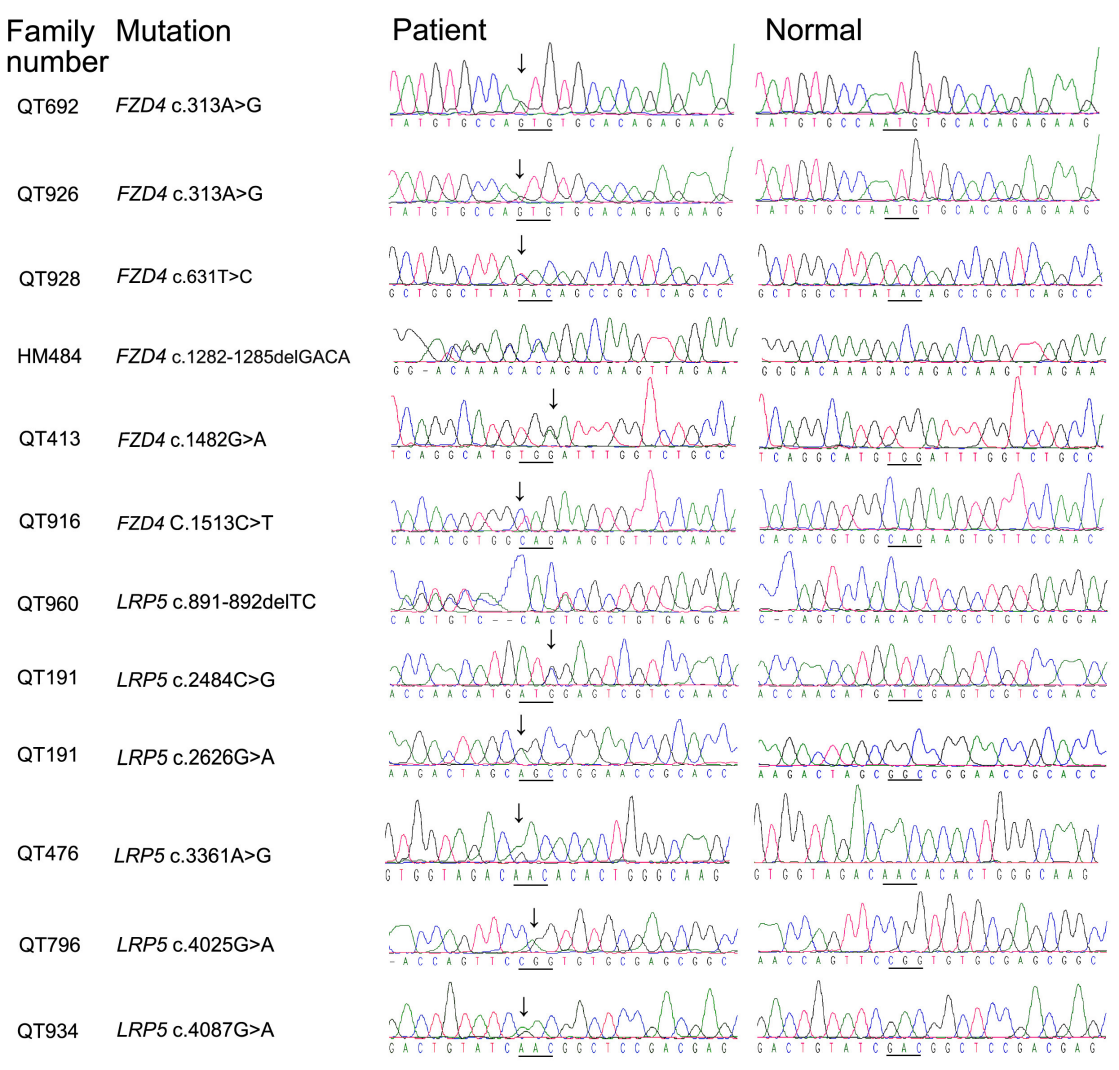
Eleven mutations identified in *FZD4* and *LRP5* genes of 49 families with FEVR. The columns from left to right display the family number, the mutation designation, and sequence chromatography from patients and normal controls.

**Figure 2:**
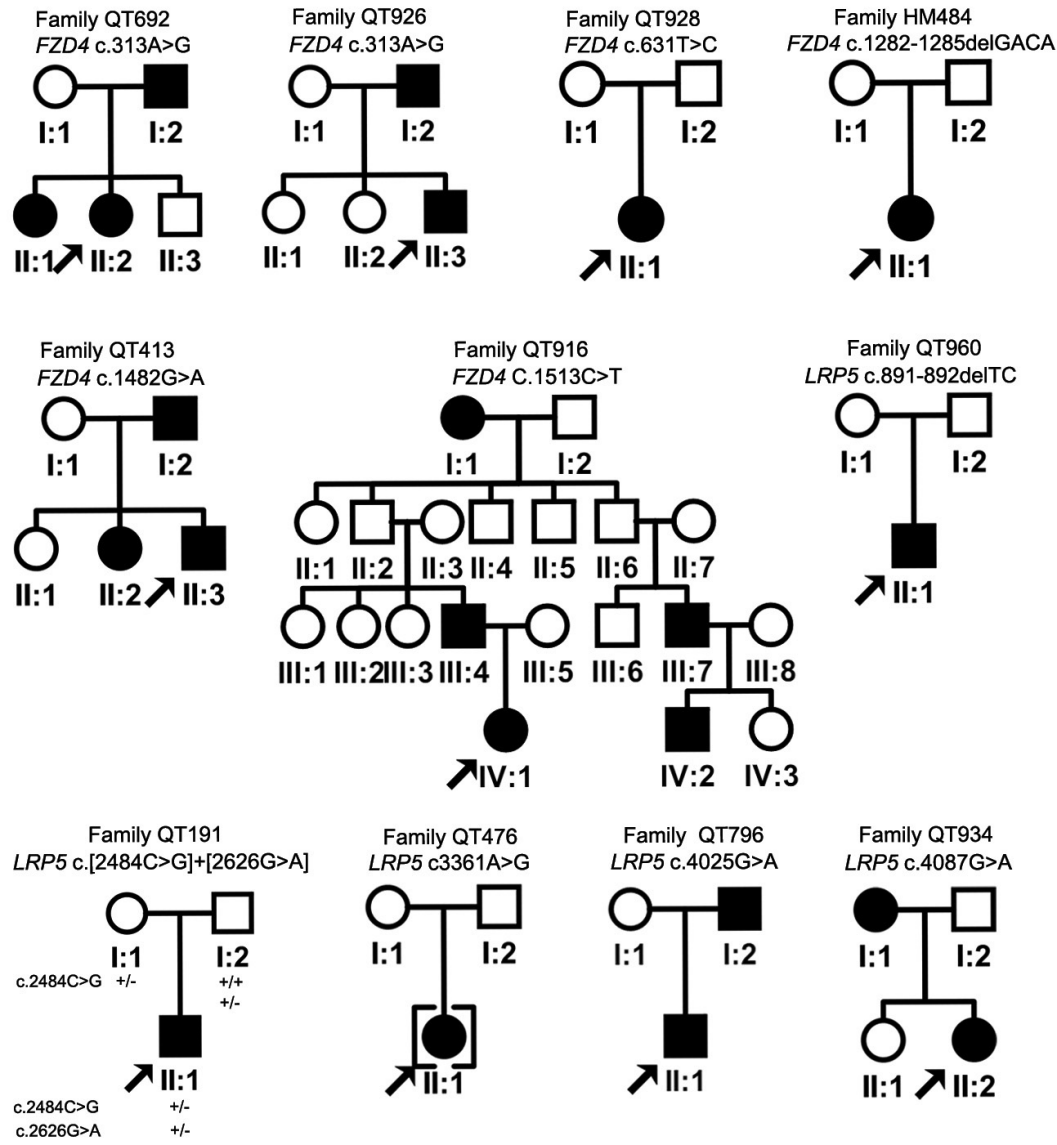
Pedigrees of 11 families with *FZD4* or *LRP5* mutations. A + sign represents a normal allele, and a - sign indicates a variant. The proband in family QT191 had compound heterozygous mutation, while his mother had a heterozygous c.2484C>**G**: variant and his father had a heterozygous c.2626G>A variant. The squares brackets around II:1 in family QT476 indicated an adopted proband.

Eleven heterozygous mutations (Figure 1), including eight novel and three known mutations, were identified in 11 of the 49 (22.4%) families with FEVR, including five *FZD4* mutations in six families and six *LRP5* mutations in five families (Table 1). Of the 11 families with *FZD4* and *LRP5* mutations, six had a familial history of FEVR, and five were isolated cases (Figure 2). Two families had the same *FZD4* mutation, and one family had compound heterozygous mutations in *LRP5*.

**Table 1:** Mutations identified in the FZD4 and LRP5 genes of the families with FEVR.

Family number	Gene/exon	DNA change	Allele status	Protein change	Computational prediction		Occurrence in		Note
					Blosum 62	PolyPhen	families	controls	
QT692,QT926	*FZD4/2*	c.313A>G	Hetero	p.Met105Val	5→-2	probably damaging	2/49	N/A	Known [25]
QT928	*FZD4/2*	c.631T>C	Hetero	p.Tyr211His	7→2	benign	1/49	0/96	Novel
HM484	*FZD4/2*	c.1282–1285delGACA	Hetero	p.Asp428SerfsX2	N/A	N/A	1/49	0/96	Novel
QT413	*FZD4/2*	c.1482G>A	Hetero	p.Trp494*	N/A	N/A	1/49	0/96	Novel
QT916	*FZD4/2*	c.1513C>T	Hetero	p.Gln505*	N/A	N/A	1/49	N/A	Known [18]
QT960	*LRP5/5*	c.891–892delTC	Hetero	p.Arg298Leu fsX2	N/A	N/A	1/49	0/96	Novel
QT191	*LRP5/11*	c.2484C>G	Hetero	p.Ile828Met	4→1	probably damaging	1/49	0/96	Novel
QT191	*LRP5/12*	c.2626G>A	Hetero	p.Gly876Ser	6→0	probably damaging	1/49	0/96	Novel
QT476	*LRP5/15*	c.3361A>G	Hetero	p.Asn1121Asp	6→1	possibly damaging	1/49	N/A	Known [27]
QT796	*LRP5/19*	c.4025G>A	Hetero	p.Arg1342Gln	5→1	probably damaging	1/49	0/96	Novel
QT934	*LRP5/19*	c.4087G>A	Hetero	p.Asp1363Asn	6→1	probably damaging	1/49	0/96	Novel

Abbreviations: Hetero: Heterozygous, N/A: Not available.

**Figure 3:**
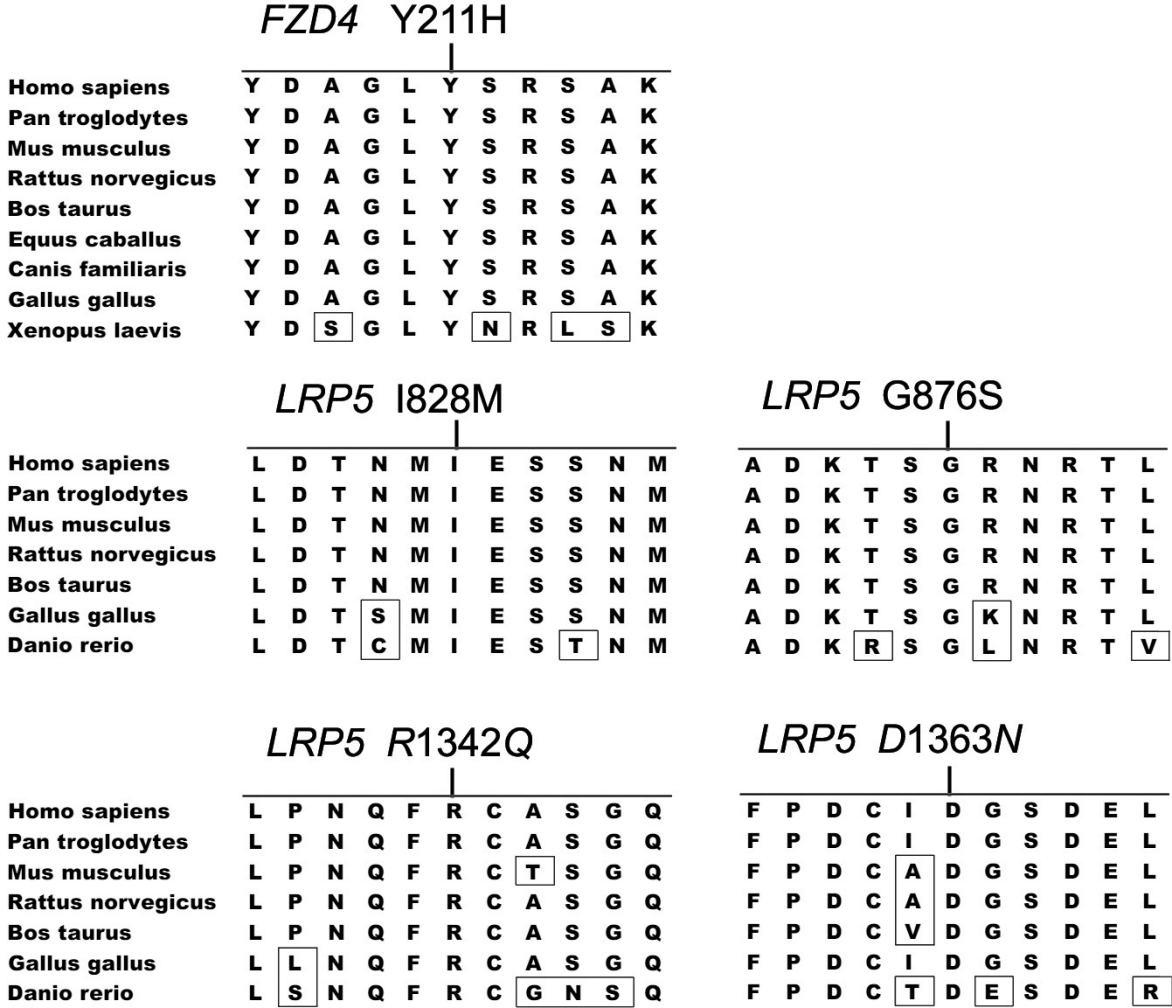
Protein alignment for the novel missense mutations identified in *FZD4* and *LRP5*. Nonconserved amino acid residues are boxed. The residues with mutations are highly conserved. *FZD4* orthologs included *Homo sapiens* (NP_036325.2), *Pan troglodytes* (XP_001175326.1), *Mus musculus* (NP_032081.2), *Rattus norvegicus* (NP_072145.1), *Bos taurus* (NP_001193198.1), *Equus caballus* (XP_001489854.1), *Canis familiaris* (XP_848753.1), *Gallus gallus* (NP_989430.1), and *Danio rerio* (XP_002664771.1). The *LRP5* orthologs are from *Homo sapiens* (NP_002326.2), *Pan troglodytes* (XP_508605.2), *Mus musculus* (NP_032539.1), *Rattus norvegicus* (NP_001099791.2), *Bos taurus* (XP_614220.3), *Gallus gallus* (NP_001012915.1), and *Danio rerio* (NP_001170929.1).

Of the 11 mutations, seven were missense, two were nonsense, and two were frameshift deletions. The eight novel mutations were not detected in 192 chromosomes of 96 normal controls. All five novel missense changes affected evolutionarily conserved residues (Figure 3), and four of the five were predicted to be pathogenic (Table 1). The cosegregation of the mutation in additional family members who were screened is shown in Table 2.

**Table 2:** Clinical information on the patients with *FZD4* or *LRP5* mutations.

Family number	ID/Sex/Age	Mutation (gene/DNA)	Best vision (right; left)	Main phenotypes	
				Right eye	Left eye
QT692	II:2/F/20y	FZD4/c.313A>G	0.3; 0.3	IBPV	RD, AZ, PFP, NV
	I:2/M/52y	FZD4/c.313A>G	1.0; 1.0	IBPV	IBPV
	II:1/F/21y	FZD4/c.313A>G	1.0; 1.0	IBPV	IBPV
QT926	II:3/M/5y	FZD4/c.313A>G	N/A	IBPV	TDOD
	I:2/M/36y	FZD4/c.313A>G	FC; 1.0	IBPV, AZ, FPF	IBPV, AZ
QT928	II:1/F/4y	FZD4/c.631T>C	N/A	TDOD	TDOD, PFP
HM484	II:1/F/4y	FZD4/c.1282–1285delGACA	0.3; 0.1	STA	TDOD, PFP
QT413	II:3/M/9y	FZD4/c.1482G>A	0.02; 0.8	RFM, LD	AZ, PFP, BPV
	I:2/M/39y	FZD4/c.1482G>A	1.0; 1.0	AZ, BPV, NV	AZ, BPV, NV
	II:2/F/14y	FZD4/c.1482G>A	0.6; 0.8	RD,TDOD	STA
QT916	IV:1/F/2y	FZD4/c.1513C>T	N/A	TDOD	FPF
	III:4/M/26	FZD4/c.1513C>T	N/A	IBPV	TDOD, BPV, AZ, PE
QT960	II:1/M/2mo	LRP5/c.891–892delTC	NLP; NLP	RFM, RD,MC, FAC	RFM, RD,MC, FAC
QT191	II:1/M/5mo	LRP5/c.[2484C>G]+[2626G>A]	NLP; NLP	RFM, SCP	RFM, SCP
QT476	II:1/F/1y	LRP5/c.3361A>G	HM; HM	TDOD	RFM
QT796	I:2/M/24y	LRP5/c.4025G>A	LP; 0.2	RFM	AZ
	II:1/M/5mo	LRP5/c.4025G>A	N/A	NYS, MC, RFM	NYS, MC, RFM, SCP
QT934	II:2/F/6mo	LRP5/c.4087G>A	HM; HM	RFM	RFM
	I:1/F/30y	LRP5/c.4087G>A	0.8;0.8	IBPV	IBPV

Abbreviations: M: Male, F: Female, y: Years old, mo: Months, FC: Finger counting, HM: Hand move, LP: Light perception, NLP: No light perception, N/A: Not available, IBPV: Increased branching of peripheral vessels, RD: Retinal detachment, PFP: Peripheral fibrous proliferation, NV: Neovascularization, TDOD: Temporal dragging of optic disc, AZ: Avascular zone, FRF: Falciform retinal fold, STA: Straightening of temporal arcades, RFM: Retrolenticular ﬁbrotic mass, LD: Lens dislocation, BPV: Brushlike peripheral vessels, PE: Peripheral exudates, MC: Microcornea, FAC: Flat anterior chamber, SCP: Stretched ciliary process. NYS: Nystagmus.

### Phenotypes

**Figure 4:**
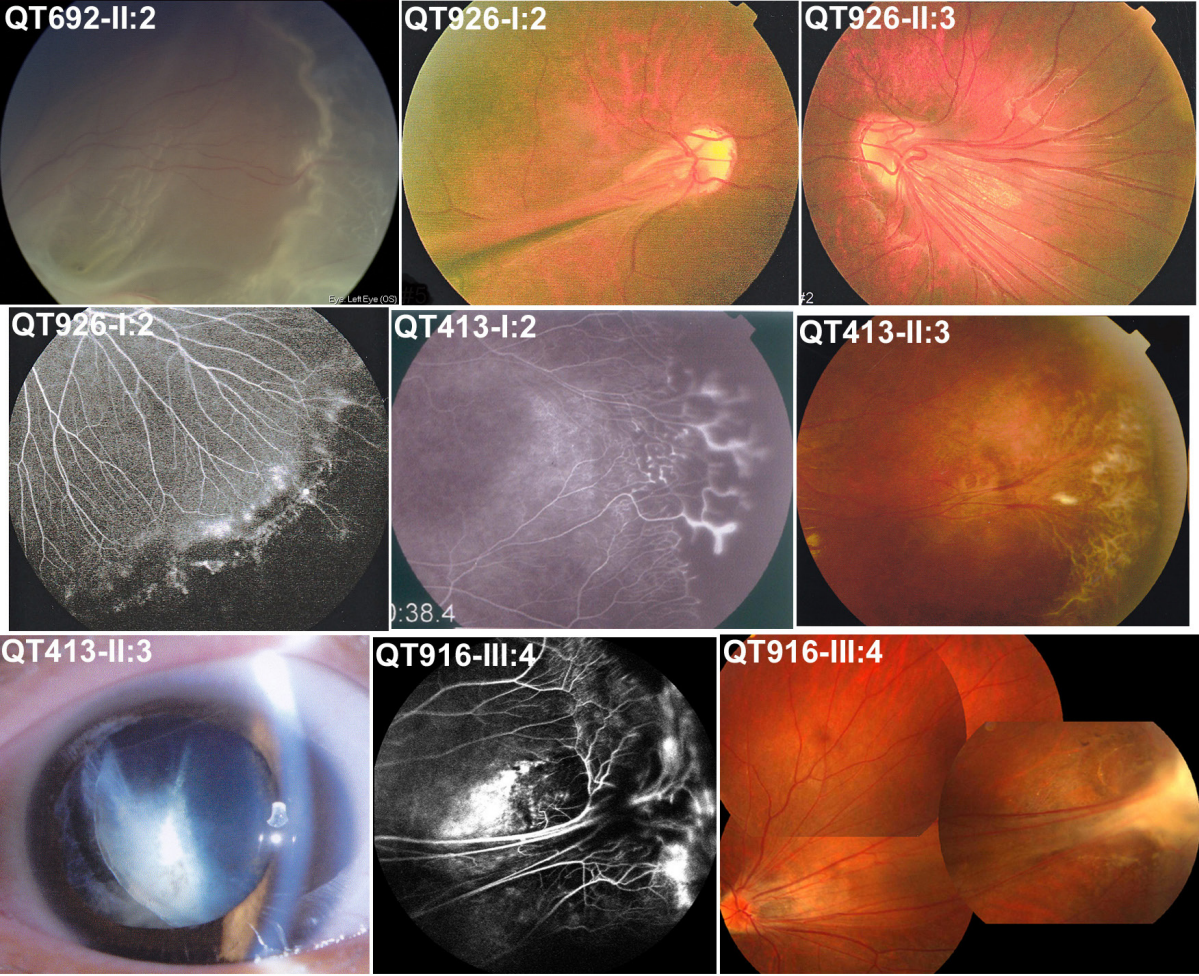
Ocular changes in affected individuals with an *FZD4* or *LRP5* mutation. The individual ID is indicated on the top left of each picture, which is the same as in Figure 2 and Table 2. Signs of FEVR included retinal detachment (top left), falciform retinal fold (top middle), temporal dragging of optic disc (top right), peripheral avascular zone and brush-like peripheral vessels (middle left), shell-like peripheral vessel terminatio and neovascularization (center), peripheral fibrovascular proliferation (middle right), lens dislocation (bottom left), peripheral exudates (bottom middle), temporal dragging of optic disc, and peripheral fibrous proliferation (bottom right).

All 11 probands and their affected relatives with *FZD4* or *LRP5* mutations had ocular changes typical of FEVR (Table 2). Individuals with mutations may be asymptomatic or blind, with visual acuity ranging from normal to no light perception. Fundus changes varied significantly in the different patients, with mildly affected individuals showing brush-like or increased branching of the peripheral vessels, peripheral avascular zone, peripheral fibrous proliferation, and/or straightening of the temporal arcades (Figure 4). These signs were also prevalent in the “healthy eye” of the probands or affected relatives, especially under examination with fluorescein angiography. The affected eyes of the probands and the relatives showed more severe ocular changes, including temporal dragging of the optic disc, falciform retinal folds, neovascularization, exudates, tractional retinal detachment, and/or retrolenticular ﬁbrotic masses.

## Discussion

In this study, 11 mutations in *FZD4* and *LRP5* were detected in 11 families with FEVR but were not present in 96 normal individuals. Based on the results of segregation analysis in the family members and the functional prediction of the mutations, these mutations appear to be the cause of FEVR in the Chinese patients.

The phenotypes of all the patients with *FZD4* or *LRP5* mutations were closely related to the developmental anomalies observed in the retinal vessels and the resulting complications. However, we documented great variability in the clinical signs between the right and left eyes of the same patient, among different affected members of the same family, and between different families. We have not identified specific phenotypes that can establish a genotype-phenotype correlation for different mutations in the same gene or for mutations in different genes.

So far, several mutations have been identified in the *FZD4* and *LRP5* genes of patients with FEVR. Mutations in *FZD4* have been detected in 5%–40% of families with FEVR [18,19,25–29], whereas those in *LRP5* have been identified in 12%–18% of families [18,26,27]. In our study, the *FZD4* and *LRP5* mutations were identified in 11 of 49 families, in which *TSPAN12* mutations have been excluded by our previous study [21]. We detected *FZD4* mutations in 9.6% (5/52) of families with FEVR and *LRP5* mutations in 11.5% (6/52) if the three families with *TSPAN12* mutations are taken into account [21]. In summary, mutations in *FZD4*, *LRP5*, and *TSPAN12* were not detected in a large proportion of families (73.1%, 38 of the 52 families) in our case series. For those families in which we failed to detect mutations, a small number might have mutations in the intronic or regulatory regions of these genes, which could not be detected with the strategies used in this study. Mutation in the *NDP* gene has been excluded in the remaining 38 families in our recent study [30]. It is more likely that additional genes involved in FEVR have yet to be discovered. Other components in the Wnt/Norrin signaling pathway might be potential candidates for further screening since all four known FEVR causative genes encode proteins involved in this pathway. Moreover, the samples in which mutations were not identified will be good targets for identifying additional FEVR genes with next-generation sequencing and exome sequencing in the near future.

## Appendix 1.

Primers used for PCR amplification and sequencing of *FZD4* and *LRP5* Abbreviations: E: Exon, F: Forward, R: Reverse, bp: Base pair. *FZD4*-E2 was amplified in three overlapping segments A, B, and C. Primer sequences for *LRP*5-E4 obtained from Gong et al. [31]. Sequencing primer for *LRP5*-E10-F is another primer TTCCTCCTCACCTGCTG. To access the data, click or select the words “Appendix 1.” This will initiate the download of a compressed (pdf) archive that contains the file.
